# The effect of intranasal oxytocin on visual processing and salience of human faces

**DOI:** 10.1038/s41398-020-00991-3

**Published:** 2020-09-19

**Authors:** Daniel Hovey, Louise Martens, Bruno Laeng, Siri Leknes, Lars Westberg

**Affiliations:** 1 Department of Pharmacology, Institute of Neuroscience and Physiology, The Sahlgrenska Academy, University of Gothenburg, Gothenburg, Sweden; 2 Department of Psychiatry and Psychotherapy, University of Tübingen, Tübingen, Germany; 3 Max Planck Institute for Biological Cybernetics, Tübingen, Germany; 4 Department of Psychology, University of Oslo, Oslo, Norway; 5 RITMO Centre for Interdisciplinary Studies in Rhythm, Time and Motion, Oslo, Norway

**Keywords:** Neuroscience, Physiology

## Abstract

The mechanisms underlying the role of oxytocin (OT) as a regulator of social behavior in mammals are only partly understood. Recently, it has been proposed that OT increases the salience of social stimuli. We carried out a randomized, double-blind, cross-over study of the effects of OT on binocular rivalry, a visual phenomenon underpinned by the interplay of excitation and inhibition in the cortex. A final sample of 45 participants viewed images of social stimuli (faces with different emotional expressions) and non-social stimuli (houses and Gabor patches). We demonstrate a robust effect that intranasal OT increases the salience of human faces in binocular rivalry, such that dominance durations of faces are longer—this effect is not modulated by the facial expression. We tentatively show that OT treatment increases dominance durations for non-social stimuli. Our results lend support to the social salience hypothesis of OT, and in addition offer provisional support for the role of OT in influencing excitation-inhibition balance in the brain.

## Introduction

The neuropeptide oxytocin (OT) has long been implicated in the regulation of social behavior in mammals, including humans^1^. The exact role of OT in this regulation, and the mechanisms behind it, are only partly understood. The picture is complicated by the wealth of studies reporting varying results of the administration of intranasal OT, including both prosocial and antisocial effects^2,3^. Several hypotheses have been proposed to explain the influence of OT on social behavior.

The social salience hypothesis suggests that OT increases the salience of social cues, which in turn can increase or modulate the influence of context and inter-individual factors on social behavior^4,5^. While the effects of exogenously administered OT are not confined solely to social behavior or social cognition, many studies have indicated that exogenously administered OT affects for example, memory for faces^6^, detection of social words^7^, and learning from social feedback^8^. Such effects have been observed for both positive and negative stimuli^9–13^. While individual studies have reported selective effects on stimuli with either positive or negative valence, taken together evidence is in line with a general increase of the salience of social stimuli, regardless of the valence of those stimuli. Thus, while OT presumably has several different effects on social cognition, the social salience hypothesis would explain much of the results of studies on exogenously administered OT.

Binocular rivalry is a visual phenomenon that occurs when dissimilar images are presented to different eyes (e.g., a face to one eye and a house to the other). The images then compete for visual awareness, i.e. the image that the viewer consciously perceives (dominant percept) is one of the presented images, while the other one is suppressed and unseen. After a few seconds, the suppressed image become dominant and the previously dominant image becomes suppressed. This results in transitions between which image is consciously perceived, and these transitions may be instant or contain a mix of the two images (piecemeal percept). This phenomenon has been extensively used to study conscious visual awareness, where a potentially salient visual stimulus can be dominant or suppressed for relatively long periods of time (several seconds)^14^. Thus, studies using binocular rivalry may yield insights regarding information processing and awareness—what enters our conscious minds, and what influences this salience? Previous studies have investigated binocular rivalry in relation to altered social cognition in psychiatric disorders, and shown reduced salience (shorter dominance durations) for positive social cues in the form of smiling faces in social anxiety disorder^15^, increased initial salience (initial dominant percept) for fearful faces in anxiety disorders^16^, and altered binocular rivalry alternation rates for emotional faces with increased depressive symptoms^17,18^. Simple characteristics of the stimuli, such as contrast, spatial frequency, and color, can also influence their strength^19,20^. In addition, the strength of a stimulus can be influenced by what that stimulus is associated with, such as neutral faces paired with negative gossip being more dominant than those paired with positive or neutral gossip^21^—demonstrating clear top-down influences on visual consciousness.

Classical accounts of the neural underpinnings of binocular rivalry proposed a reciprocal and fluctuating lateral inhibition of the visual cortices^22^. Later models incorporated inhibitory and excitatory components, as well as top-down influences^14,23,24^, such as attentional aspects, and meaningfulness of the stimulus. While the specific mechanics of this top-down influence remain to be determined, the reciprocal action in the primary visual cortices is believed to depend on the balance of excitation^23,25^ and inhibition^26,27^. A link between binocular rivalry and excitation-inhibition balance is also supported by findings in individuals with autism spectrum disorder (ASD)^28–30^, where that balance is thought to be disturbed^31,32^.

Accordingly, binocular rivalry offers a so far untapped opportunity to investigate how OT may influence the salience of social cues in humans. Using for the first time binocular rivalry in combination with intranasal OT treatment, and in line with the social salience hypothesis, we hypothesized that the dominance of a face (i.e., a visual stimulus rich in social cues) would increase when exogenous OT is administered, and that this effect should be specific to social stimuli, as well as independent of the affective valence of the face.

## Methods

### Participants

A total of 50 healthy male volunteers (mean age = 27.9 ± 6.2 years) were recruited through advertising online and at the local campus, and were monetarily compensated with 300 NOK (approximately 34 USD). All participants had normal visual acuity, either unaided or with correction, and reported no eye conditions (such as amblyopia, strabismus, or diplopia) or any pertinent medical conditions. Color blindness was screened for using Ishihara color plates. Following a description of the aim of the study and the procedures involved, written consent was obtained. The study was approved by the Regional Ethics Committee (2009/208/REK sør-øst C).

### Drug protocol

OT (Syntocinon®, Novartis) and placebo (normal saline, 0.9%, Miwana) were administered in the form of nasal spray in identical conventional pump-actuated bottles (five puffs in each nostril). OT was administered in a dose of 40 IUs, as in our previous study^33^. The study used a randomized, double-blind, placebo-controlled, cross-over design, such that all subjects received both OT and placebo treatment over the course of two visits on separate days. Using Latin squares, participants were randomly assigned to receive either OT or placebo on their first visit. The participants as well as the experimenter were blinded as to whether they received OT or placebo on their separate visits. The average length of time between visits was 5.73 days (range 3–12 days).

Participants were verbally instructed to self-administer the intranasal spray in each nostril, and were explicitly instructed to sniff well and to not tilt their head back during the administration. At the end of each visit, the participants were asked whether or not they thought they received OT, and did not perform better than chance (57% correct; *χ*^2^(1) = 1.089; *p* > 0.05). Since the test described below was part of a battery of four brief tests, participants were randomized for the order of these tests, and this order was then kept constant between sessions. The test described below would thus take place ~30–60 min after administration of OT or placebo.

### Stimuli

**Fig. 1:**
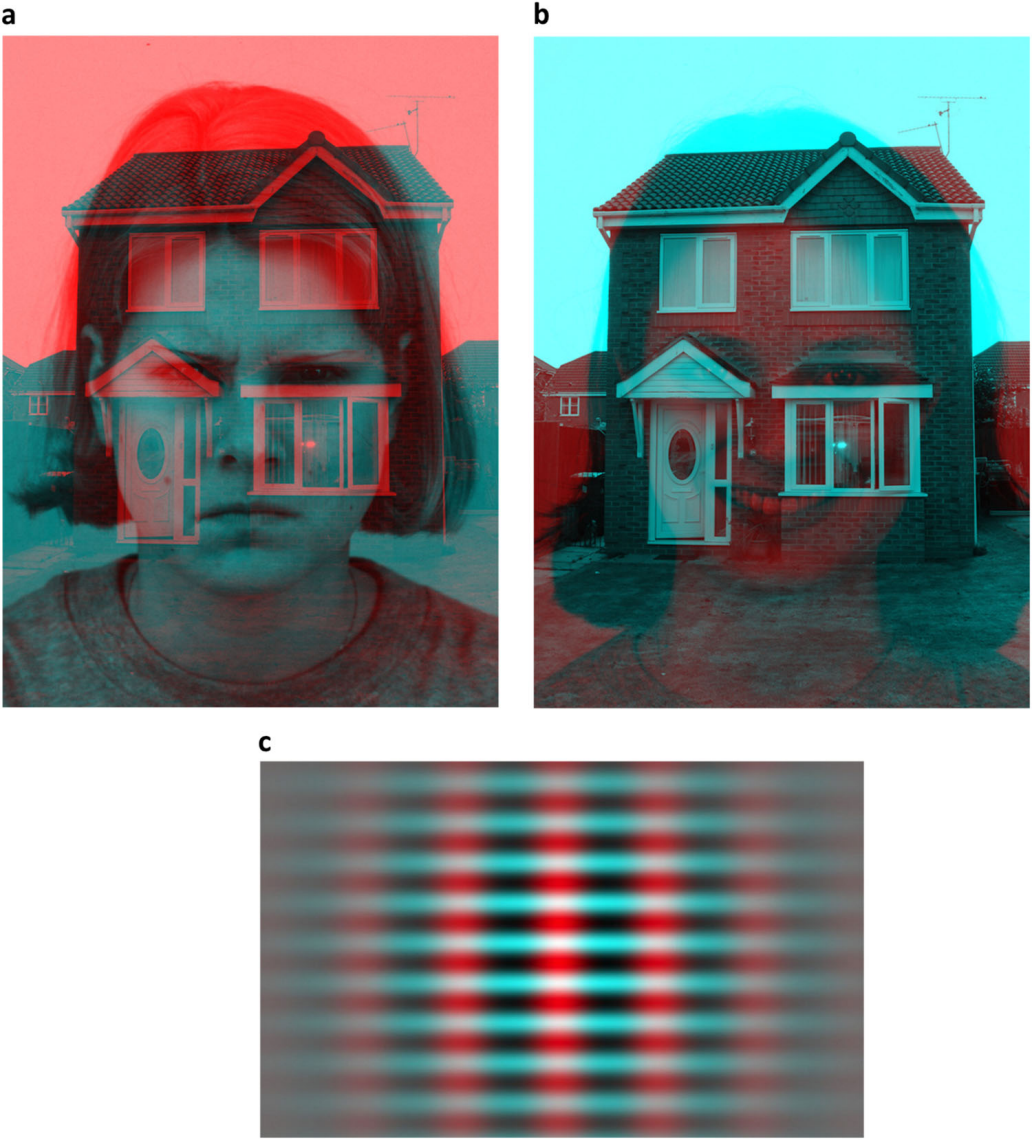
Anaglyph examples. **a** Face/house anaglyph. **b** Face/house anaglyph with inverted colors. **c** Gabor anaglyph.

During each of the two visits, participants viewed a series of 30 stationary anaglyphs—please see Fig. 1 for examples^34^. Of these, 28 consisted of an image of a female face (sourced from the Karolinska Directed Emotional Faces) and a superimposed house image (public domain image available online), where the face would be either red or cyan, and the house would have the opposite color. Half of the anaglyphs had a red face and half had a cyan face. The face displayed one of six emotional expressions—joy, anger, sadness, surprise, fear, or disgust—or had a neutral expression. Thus, four anaglyphs out of the 28 would display each expression. Two final anaglyphs consisted of Gabor patches, i.e., intersecting vertical and horizontal low spatial frequency lines. Gabor patches are thus visual stimuli with low complexity and no social information. One of the pictures had red vertical lines and cyan horizontal lines, and one had the opposite arrangement. See Fig. 1 for examples of face/house and Gabor anaglyphs. The anaglyphs were presented using SMI Experiment Center (SensoMotoric Instruments, RED500, SMI^©^ Berlin, Germany) on a stationary computer. The anaglyphs were presented at an approximate viewing angle of 38 degrees, with participants positioned at a distance of 70 cm from the computer screen.

### Experimental procedure

Each participant was provided with colored anaglyph glasses (red and cyan), and received instructions prior to a brief practice trial. Each experimental run consisted of the 28 face/house anaglyphs being presented in sequence to the participant. Each anaglyph would be presented for 30 s, with one anaglyph directly following the next. This sequence of 28 anaglyphs were then followed by a brief one minute break, after which the participant then viewed the two Gabor anaglyphs, which would be presented for 60 s each. The participant self-reported dominant or piecemeal percepts by pressing one of three buttons to indicate what they perceived. Button presses were registered with a single time stamp, regardless of how long the button was pressed. While viewing the face/house anaglyphs, the left button would indicate face dominance, the right one house dominance, and the middle one piecemeal percept. During viewing of the Gabor anaglyphs, the left and right buttons would indicate dominant red or cyan percept, respectively, and the middle one piecemeal percept.

### Data analysis and statistics

Data retrieved from BeGaze software (SMI^©^ Berlin, Germany) contained timed key presses for each of the separate anaglyphs. Data were extracted using an in-house script written in Python 3.4 (see Supplementary Material).

We extracted durations of dominance (ms) of uninterrupted percepts for all anaglyphs, categorized as face, house, and Gabor dominance durations, respectively. The time before the initial button press for each anaglyph was excluded, since there was no way to accurately determine whether the indicated percept before initial key press was in fact what the participant was seeing, or if it was caused by bleed-over from the previous anaglyph, or simply reaction time latency. Similarly, the time following the ultimate button press for each anaglyph was also excluded, to avoid artificially shortened durations. All dominance duration means were calculated following an initial exclusion of dominance durations shorter than 250 milliseconds or longer than the mean plus three standard deviations across all participants for the two types of anaglyph (face/house and Gabor, respectively). We extracted average durations of face dominance duration for all separate emotions (seven variables per session), and also averaged durations across all emotions for an overall mean duration of face dominance duration (one variable per session). House dominance durations were averaged across all emotions (one variable per session). Gabor dominance was averaged across both dominance percepts (one variable per session).

We also calculated alternation rates for face/house anaglyphs and Gabor anaglyphs, respectively, since this has been previously used to investigate excitation-inhibition balance^30^. An alternation was defined as a complete transition from one dominant percept to another, and alternation rate was quantified as the number of alternations per 30 s.

Five participants were excluded from statistical analyses for consistently displaying no meaningful rivalry (i.e., less than two key presses for more than 50% of stimuli in either or both of the sessions) or misunderstanding the instructions. This yielded a final sample of 45 participants. In order to ensure good quality data, results from individual stimuli were excluded if they contained less than two key presses, leading to a data loss of 3.9% from the *n* = 45 sample.

The data were analyzed using SPSS 23. We utilized repeated measures analysis of variance (RM-ANOVA) to analyze average dominance durations between different dominant percepts (face, house, and Gabor), where percept type and drug were within-subject factors. RM-ANOVA was also used to investigate the modulation of dominance durations for face percepts and face predominance by emotional content, where drug and emotion were within-subject factors. Post-hoc tests were paired t-tests. Alternation rates were tested using paired *t*-tests for the average rates across face/house and Gabor anaglyphs, respectively. All tests were two-tailed.

We did not conduct a specific power analysis prior to data collection for the binocular rivalry task. With 45 people per group in a between-subjects study, for a two-tailed contrast (*t*-test) G*power estimates 80% power to detect a medium effect, *d* = 0.6. However, in within-subjects studies of drug effects on behavioral task outcomes, correlations between drug and placebo are typically 0.6 or higher^35–39^. In our data, the correlation coefficient of average dominance durations of faces, houses, and gabors between drug conditions ranged from *r* = 0.687 to *r* = 0.829. Assuming *r* = 0.6 between oxytocin and placebo condition measures of binocular rivalry based on previous literature only, our within-subjects study is at least as sensitive as a between-subjects study with 75 participants per group, i.e. has 80% power to detect *d* = 0.46. This means that even considering the boost in statistical power yielded by our design, the study lacked the sensitivity to detect small effects.

## Results

Please see Table 1 for all pairwise comparisons mentioned below.

**Table 1:** Pairwise comparisons for percepts/anaglyphs.

Variable	Percept/Anaglyph	Placebo	Oxytocin	df	*t*	*p*
Average dominance duration (ms)	Face—Combined	2363 ± 125	2707 ± 158	44	3.86	0.0004
	Face—Joy	2399 ± 163	2650 ± 181	43	1.31	0.2
	Face—Anger	2358 ± 143	2768 ± 216	43	1.96	0.057
	Face—Sadness	2327 ± 142	2785 ± 190	43	3.41	0.001
	Face—Surprise	2431 ± 139	2674 ± 165	43	1.68	0.1
	Face—Fear	2340 ± 171	2728 ± 219	44	1.87	0.068
	Face—Disgust	2152 ± 125	2719 ± 205	44	3.2	0.003
	Face—Neutral	2561 ± 193	2618 ± 169	42	0.29	0.78
	House	2639 ± 105	2806 ± 134	44	1.69	0.098
	Gabor	1658 ± 66	1775 ± 91	42	1.86	0.069
Average piecemeal duration (ms)	Face/House	2601 ± 124	2677 ± 184	44	0.41	0.68
	Gabor	1523 ± 113	1583 ± 126	41	0.42	0.68
Average alternation rate	Face/House	3.6 ± 0.4	3.9 ± 0.3	44	1.26	0.22
(switches/30 s)	Gabor	10.5 ± 0.6	9.6 ± 0.6	42	1.45	0.16

All means presented with standard error of the mean.

### Dominance durations

**Fig. 2:**
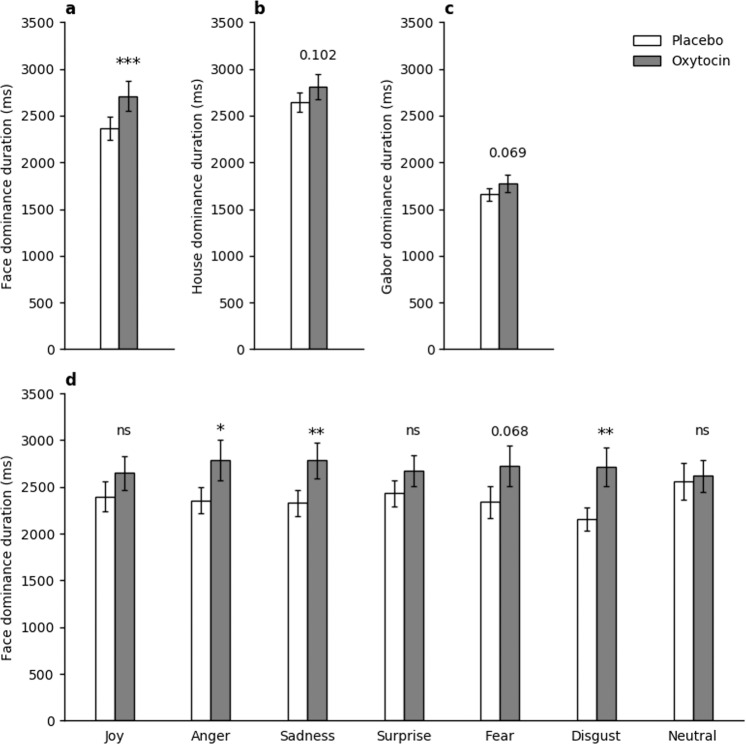
Dominance duration results. **a**–**c** Dominance durations for faces, houses, and Gabor patches, respectively. **d** Dominance durations for faces by emotion. Means ± standard error of the mean. PLC: placebo; OXT: oxytocin.

Average dominance durations (ms) of uninterrupted percepts were calculated for face (across all emotions), house, and Gabor percepts. Using RM-ANOVA with a 2 (oxytocin/placebo) × 3 (face, house, or Gabor percept) design, we found a significant main effect of drug (*F*(1,42) = 10.64; *p* = 0.002; *ηp*^2^ = 0.202) and of percept (*F*(2,41) = 45.74; *p* < 0.001; *ηp*^2^ = 0.691), as well as an interaction between drug and percept (*F*(2,41) = 3.50; *p* = 0.039; *ηp*^2^ = 0.146). Subsequent post-hoc analyses revealed that compared to the placebo treatment, OT significantly increased face dominance durations for all emotions combined (*t*(44) = 3.86; *p* < 0.001; *ηp*^2^ = 0.253; Fig. 2a).

The similar combined measure for house dominance durations was on the border of statistical significance for OT treatment increasing dominance durations for houses as well (*t*(44) = 1.69; *p* = 0.098; *ηp*^2^ = 0.061; Fig. 2b), compared to placebo. Similarly, Gabor dominance durations showed a trend approaching significance for being increased in the OT condition compared to the placebo condition (*t*(42) = 1.86; *p* = 0.069; *ηp*^2^ = 0.076; Fig. 2c).

Dominance durations for faces were also averaged separately for the different emotions. RM-ANOVA analysis with a 2 (oxytocin/placebo) × 7 (emotions) was performed, with Mauchly’s test of sphericity indicating that sphericity was violated for drug × emotion (*W* = 0.221; *p* < 0.001). The degrees of freedom were thus corrected using Huyhn-Feldt estimates of sphericity. There was no significant interaction between drug and emotion of the face perceived on dominance durations (*F*(5,200) = 0.975; *p* = 0.431). The face dominance durations were higher with oxytocin for all emotional face categories. Post-hoc paired *t*-tests comparing oxytocin and placebo reached significance for sadness and disgust. The mean difference in dominance durations was least apparent for the neutral condition (Fig. 2d).

There were no statistically significant differences in piecemeal durations, for face/house stimuli or for Gabor stimuli (all *p* > 0.05).

### Alternation rates

We included alternation rates as an outcome since this has been previously utilized in order to investigate excitation-inhibition balance with binocular rivalry^30^. There was, however, no significant difference between intranasal oxytocin and placebo with regards to alternation rate between the face and house percept, defined as alternations/30 s (*t*(44) = 1.18; *p* = 0.25). There was also no significant difference between the oxytocin and placebo condition with regards to alternation rate between dominant Gabor percepts, defined as switches/30 s (*t*(42) = 1.45; *p* = 0.16).

## Discussion

To our knowledge, this is the first study to utilize binocular rivalry to investigate the effects of intranasal OT on visual processing. Longer dominance durations for faces during binocular rivalry confirmed our hypothesis that intranasal OT increases the salience of faces. The higher effect size for faces rather than non-social stimuli is congruent with the social salience hypothesis^4^; we however, did not find that emotional content significantly modulated the response to OT administration. Our data thus provides support for the idea that OT increases the salience of social signals of positive and negative affective valence. Nevertheless, we also found non-significant but intriguing trends that intranasal OT increases the dominance durations for both Gabor and house percepts—both non-social in nature. We did not find any significant differences in alternation rates between placebo and oxytocin for the face/house anaglyphs, nor for the Gabor anaglyphs.

The increase in salience across valences is in line with studies where the outcome measure can be assumed to depend on the salience of stimuli, for instance showing that intranasal OT increases memory retention of faces^6,12^ and emotion recognition accuracy^11,40^. The role of OT in face and emotion recognition has been stressed by the above mentioned pharmacological as well as genetic studies^41–44^; however, other studies report incongruent results^39^. We would argue that our results are novel in their methodological approach, and lend support to the social salience hypothesis, through a psychophysiological measurement.

There are several potential mechanisms by which OT may influence rivalry dynamics and the processing of visual social information. Firstly, OT may have increased face dominance during binocular rivalry via attentional mechanisms^45–48^. Recent neuroimaging studies using intranasal OT are in line with enhanced visual attention, e.g., enhanced early visual activity for faces^49^, and increased functional connectivity in the nucleus basalis of Meynert, which regulates selective attention^50^. This interpretation of our results is in line with the social salience hypothesis, where attention, as guided by oxytocinergic influences on dopaminergic circuitry, is central^4^. Secondly, previous pharmacological studies on the effects of the serotonin receptor agonists psilocybin and tandospirone demonstrated increased dominance durations for Gabor patches^51,52^, suggesting that serotonin acts to modulate binocular rivalry. OT has been shown to directly influence the release of serotonin^53^, in for instance the nucleus accumbens^54^. Serotonergic signaling, therefore, constitutes a potential mediator of OT’s effects on rivalry dynamics. Thirdly, binocular rivalry is a phenomenon based on interplay between inhibitory and excitatory neurons, and there is ample evidence that OT is able to influence excitation-inhibition balance in various parts of the brain. For example, it has been implicated in the initial transient switch in γ-aminobutyric acid (GABA) receptors from depolarizing to hyperpolarizing^55–57^. OT receptor knockout mice display a decreased ratio of GABA-ergic to total presynapses in the hippocampus, and increased seizure susceptibility^58^, and OT has been shown to influence glutamate neurotransmission in brain slices of mice^59^. Intriguingly, in the sensory cortices, OT rapidly alters the excitation-inhibition balance in the auditory cortex to enable maternal behavior in female mice^60^, as well as enhance pre- and postsynaptic glutamatergic signaling in the rat olfactory bulb^61^. While we cannot, based on these three lines of reasoning, pinpoint from our data where OT acts to achieve its effects on rivalry dynamics, our results provide the support that OT does influence the excitation-inhibition balance phenomenon of binocular rivalry, by top-down effects and/or directly in the visual cortex. In addition, these proposed mechanisms may in part explain the fact that we see strong trends towards increased dominance durations for non-social stimuli as well.

Our tentative findings on non-social stimuli are congruent with previous literature indicating that the effects of OT are not restricted to only social contexts, as it may mediate for instance approach and avoidance behavior in non-social contexts as well^62^. The approach-avoidance hypothesis of OT has not been specifically addressed here, and our non-social stimuli were arguably simplistic. This makes it difficult to draw any firm conclusions on how our findings may relate to this hypothesis, but provides an intriguing avenue of further research.

OT administration in mammals has been shown to rescue social behavior deficits^63^. Hence, it is not surprising that OT has been suggested as a possible treatment for social impairments in several psychiatric and neurological diagnoses, such as developmental prosopagnosia^64^, borderline personality disorder^65^, and ASD^66^. This therapeutic option is appealing, given the relatively mild side effect profile of OT, and the ease of intranasal administration; however, the effect duration is relatively short. Substantial controversy remains about how and how much intranasally administered oxytocin reaches the brain in humans^67^, and the few human studies applying variable doses have yet to produce conclusive results^68–70^. The dose applied here (40IU) is the largest dose typically applied in human research. Studies attempting treatment with intranasal OT for individuals with ASD have had encouraging, although somewhat mixed, results^63^. Our findings encourage future studies on exogenously administered OT as a treatment for social impairments, for example, in combination with behavioral therapy to fully utilize the effect duration.

Limitations to the study include that it was not powered to detect small effects, although many intranasal oxytocin studies report small effects. Another potential weakness of our study is that it included only men. Given the well-known sex-specific idiosyncrasies of the OT system^71^, we would urge future studies to include women as well. Furthermore, this study included only female faces, and future studies would do well to investigate if early visual processing of same-sex and opposite-sex faces is different. In addition, this study did not include any measure of whether each emotional expression was recognized, which would have allowed a deeper analysis of the possible correlation between a change in early visual processing and aspects of social cognition. Lastly, the participants were not asked whether they had any conditions that may have obstructed the nasal cavity (e.g., past nasal surgery, current cold/flu), which could possibly influence the response to intranasal OT.

In conclusion, we have demonstrated that intranasal OT administration leads to altered visual processing of social stimuli, by increasing dominance durations for faces in a binocular rivalry paradigm. This is an easily administered and non-invasive test, which can be further modified to probe aspects of visual awareness, with additional pharmacological manipulations, which will allow more detailed investigations of the effects of OT on perception and processing of social stimuli. Our results lend support to the hypothesis that OT increases the salience of social stimuli, and suggests that this increased salience happens across stimuli of different valences.

## Supplementary information

Supplementary Data 1
